# Impact of Resistance Training on Skeletal Muscle Mitochondrial Biogenesis, Content, and Function

**DOI:** 10.3389/fphys.2017.00713

**Published:** 2017-09-15

**Authors:** Thomas Groennebaek, Kristian Vissing

**Affiliations:** Section for Sport Science, Department of Public Health, Aarhus University Aarhus, Denmark

**Keywords:** mitochondria, strength training, bioenergetics, mitochondrial protein synthesis, mitochondrial volume density, blood flow restricted exercise

## Abstract

Skeletal muscle metabolic and contractile properties are reliant on muscle mitochondrial and myofibrillar protein turnover. The turnover of these specific protein pools is compromised during disease, aging, and inactivity. Oppositely, exercise can accentuate muscle protein turnover, thereby counteracting decay in muscle function. According to a traditional consensus, endurance exercise is required to drive mitochondrial adaptations, while resistance exercise is required to drive myofibrillar adaptations. However, concurrent practice of traditional endurance exercise and resistance exercise regimens to achieve both types of muscle adaptations is time-consuming, motivationally demanding, and contended to entail practice at intensity levels, that may not comply with clinical settings. It is therefore of principle interest to identify effective, yet feasible, exercise strategies that may positively affect both mitochondrial and myofibrillar protein turnover. Recently, reports indicate that traditional high-load resistance exercise can stimulate muscle mitochondrial biogenesis and mitochondrial respiratory function. Moreover, fatiguing low-load resistance exercise has been shown capable of promoting muscle hypertrophy and expectedly entails greater metabolic stress to potentially enhance mitochondrial adaptations. Consequently, fatiguing low-load resistance exercise regimens may possess the ability to stimulate muscle mitochondrial adaptations without compromising muscle myofibrillar accretion. However, the exact ability of resistance exercise to drive mitochondrial adaptations is debatable, not least due to some methodological challenges. The current review therefore aims to address the evidence on the effects of resistance exercise on skeletal muscle mitochondrial biogenesis, content and function. In prolongation, a perspective is taken on the specific potential of low-load resistance exercise on promoting mitochondrial adaptations.

## Introduction

Mitochondria are intracellular organelles that play a key role in metabolism and cellular homeostasis. Consequently, mitochondrial content and function exert great influence on substrate utilization capacity and skeletal muscle health, with skeletal muscle mitochondrial abnormalities implicated in the pathology of chronic disorders such as diabetes, obesity, and peripheral arterial disease (Kim et al., 2000; Petersen et al., 2004; Rontoyanni et al., 2017).

Synthesis of new mitochondrial reticular components (i.e., mitochondrial biogenesis) has profound effect on mitochondrial content and function. Mitochondrial biogenesis is reported to be attenuated with aging, prolonged inactivity, and/or chronic disease (Rooyackers et al., 1996a,b; Abadi et al., 2009; Gram et al., 2014), while metabolic stressors inherent of exercise possess the ability to stimulate mitochondrial biogenesis. In accordance, metabolic stress inherent of endurance exercise has been demonstrated to stimulate mitochondrial biogenesis (Perry et al., 2010; Di Donato et al., 2014), which, when repeated through prolonged training (i.e., endurance training), can accumulate into adaptational changes in mitochondrial content and function (Hoppeler et al., 1985; Jacobs and Lundby, 2013). Employment of animal models and omics-based studies has improved our understanding on the molecular mechanisms underlying endurance exercise-induced mitochondrial biogenesis. Accordingly, alterations in intramuscular homeostasis (e.g., alterations in AMP, calcium, and reactive oxygen species) inferred by endurance exercise are described to exert regulatory action on specific proteins involved in transcriptional regulation of mitochondrial biogenesis, such as calcium/calmodulin-dependent protein kinase (CaMK), AMP-activated protein kinase (AMPK), and p38 mitogen-activated protein kinase (p38-MAPK) (Hawley et al., 1995; Wright et al., 2007; Combes et al., 2015).

With regards to resistance exercise, high-load resistance exercise has been demonstrated to stimulate myofibrillar protein accretion, which upon prolonged training (i.e., resistance training) can accumulate into muscle hypertrophy (ACSM, 2009). However, the necessity of conducting resistance exercise with high loads has more recently been challenged by studies demonstrating that low-load resistance exercise performed to volitional fatigue, is equally capable as high-load resistance exercise in stimulating myofibrillar accretion and muscle growth (Burd et al., 2010; Mitchell et al., 2012; Farup et al., 2015). Interestingly, several studies also suggest that resistance exercise can stimulate myocellular signaling for mitochondrial biogenesis, albeit to a relatively lesser degree than endurance exercise and depending on training status and exercise principles employed (Coffey et al., 2006; Wilkinson et al., 2008; Vissing et al., 2013). However, the impact of differentiated resistance exercise regimens on skeletal muscle mitochondrial adaptations remain rather overlooked, despite potential clinical significance. In accordance, such knowledge could be beneficial in the attempt to identify a therapeutic exercise-based strategy that enable concurrent effect on mitochondrial and myofribrillar adaptations.

Below we provide a summary on the current knowledge on how resistance exercise affects skeletal muscle mitochondrial biogenesis and whether repeated resistance exercise can enhance mitochondrial content and function. As different methodological approaches have produced discrepancies in results, some important methodological issues will also be addressed. Oppositely, while the importance of mitochondrial degradation (e.g., mitophagy) is acknowledged, this will not be addressed. The summarized knowledge is retrieved from human studies, as animal models that render voluntary resistance exercise possible are lacking. Finally, since most studies on human resistance exercise have utilized high loads, we will discuss the perspectives of using low-load resistance exercise to stimulate mitochondrial adaptations in clinical populations that may be less able to perform high-load resistance exercise.

## Acute effects of single-bout resistance exercise on muscle mitochondrial biogenesis

Mitochondria are dynamic organelles residing in reticular networks and constantly adapting to accommodate new demands by processes such as biogenesis, mitophagy, fission and fusion (Kirkwood et al., 1986), with the process of mitochondrial biogenesis so far having received most attention in exercise physiology research.

Mitochondrial biogenesis has previously been evaluated from biomarkers of mitochondrial content and/or expression of metabolic transcripts. However, it has been argued that the first approach provides information on quantity rather than biogenesis *per se* and that the second approach does not appreciate the potentially attenuating effect of exercise-induced ATP-turnover on regulation of muscle protein synthesis, thus rendering direct measurements of fractional mitochondrial protein synthesis (MitoPS) rate, necessary (Miller and Hamilton, 2012; Miller et al., 2016).

Only very few studies have investigated the effect of resistance exercise on MitoPS. These studies suggest that both MitoPS and myofibrillar protein synthesis (MyoPS) rate increase during early recovery (i.e., 0–4 h) after single-bout high-load resistance exercise in healthy individuals in the untrained state (Wilkinson et al., 2008; Donges et al., 2012). On the other hand, stimulation of MitoPS, but not MyoPS, seems to be attenuated when single-bout high-load resistance exercise is conducted in a resistance exercise-accustomed state (Wilkinson et al., 2008). Interestingly, low-load resistance exercise performed with slow and tonic contraction phase and conducted to volitional fatigue has been reported to increase MitoPS rate during both early (i.e., 0–6 h) and later (i.e., 24–30 h) post-exercise recovery as well as MyoPS rate during later recovery (i.e., 24–30 h) in healthy, young, resistance exercise-accustomed individuals (Burd et al., 2012). Consequently, resistance exercise does in fact seem able to stimulate mitochondrial biogenesis, although in a manner depending on training status and the magnitude of metabolic stress imposed.

However, it is important to note that the above-mentioned studies investigated MitoPS in the fed state, while none of these studies implemented non-exercise control experiments to preclude independent effects of dietary condition (i.e., feeding or fasting) (Vissing et al., 2005). For instance, amino acid administration has been observed to stimulate MitoPS rate (Bohe et al., 2003), which also implies a role for the amino acid-sensitive signaling protein mechanistic target of rapamycin (mTOR) in regulating MitoPS. On the other hand, the exact role of mTOR for MitoPS seems complex, since resting as well as EE-induced MitoPS is maintained despite rapamycin mediated-mTOR inhibition in mice (Drake et al., 2013; Philp et al., 2015), which indicates that regulation of MitoPS may underlie mTOR-independent signaling mechanisms. Moreover, it is also interesting to note that the anabolic effects of amino acid administration on MitoPS are suppressed when insulin release is inhibited by somatostatin (Robinson et al., 2014). Teasing out such dietary effects from those of exercise requires implementations of independent non-exercise control experiments. In fact, Robinson et al. (2017) did implement a control intervention in a study investigating the effect of resistance exercise on MitoPS (Robinson et al., 2017). In this study, the authors did not evaluate acute changes in MitoPS, but rather basal MitoPS before and after prolonged high-load resistance training in healthy young and aging cohorts. Interestingly, they observed that basal MitoPS rate was increased in resistance trained aging, but not young individuals, implying that greater sensitivity exists with increasing age.

Also noteworthy, previous human studies on the effect of resistance exercise on MitoPS, are based on infusion of stabile isotope-marked amino acids or alfa-keto acids. The inherent invasive nature and demand for continuous infusion limits this approach to information on proteins with relatively fast turnover rate (i.e., hours), while not allowing for investigation on the synthesis rate of proteins with slower turnover rate (i.e., days-weeks) and/or cumulative exercise training effects (Miller et al., 2015). Furthermore, intravenous administration of amino acids may in itself or indirectly, due to stimulation of insulin secretion, confound readings of protein synthesis (Bohe et al., 2003; Robinson et al., 2014), at least when using quantities inherent of the flooding dose technique (Jahoor et al., 1992; Smith et al., 1998). More recent methodological advances in mass spectrometry have reintroduced the use of the stable isotope, deuterium oxide (D_2_O), allowing these limitations to be circumvented. In accordance, D_2_O is orally administered, and rapidly equilibrates throughout body water compartments and facilitates intrinsic labeling of amino acids and nucleic acids (Dufner et al., 2005; Busch et al., 2006). This allows for measurements of MitoPS over prolonged periods of time, while simultaneously avoiding intravenous administration of amino acids (Robinson et al., 2011). Consequently, it reduces the need for complex decisions on dietary standardization, diurnal rhythm, or invasive procedures inherent of single-bout experiments. Yet, whereas such methodological advances can provide added insight on mitochondrial biogenesis, it is not directly telling on more permanent adaptations in mitochondrial content.

## Effect of prolonged resistance training on muscle mitochondrial content

Multiple analytical approaches are available for assessment of mitochondrial content. Among these, electron microscopy-based determination of mitochondrial volume density is regarded as the golden standard (Larsen et al., 2012). However, the studies utilizing electron microscopy to deduce resistance training-induced adaptational changes in mitochondrial volume density are few and characterized by methodological constraints, such as low sample size and absence of control experiments. These methodological constraints notwithstanding, two previous studies utilizing electron microscopy surprisingly suggest a dilution of mitochondrial volume density to occur following high-load resistance training in young men (MacDougall et al., 1979; Luthi et al., 1986).

Most studies on the effect of resistance training on mitochondrial content rely on utilization of citrate synthase (CS) activity as a biomarker, because CS activity has been shown to exhibit a degree of association with mitochondrial volume density determined by electron microscopy, while simultaneously offering a significantly less time-consuming approach (Larsen et al., 2012; Meinild Lundby et al., 2017). However, results from studies on high-load resistance training in young untrained individuals that employ measures of CS activity are somewhat equivocal, with most studies reporting unaltered CS activity (Tesch et al., 1990; Wang et al., 1993; Wilkinson et al., 2008; Porter et al., 2015), whereas other studies report either decreased (Kon et al., 2014) or increased (Tang et al., 2006) CS activity. A similar discordance is evident from studies evaluating the effect of high-load resistance training on single fiber mitochondrial content by use of histochemical staining for succinate dehydrogenase (Ploutz et al., 1994; Chilibeck et al., 1999; Green et al., 1999; Bell et al., 2000).

In conclusion, while these divergent results may in part adhere to the degree of muscle hypertrophy achieved (Tang et al., 2006), they are also the product of a number of small-scale studies, with only few to include control experiments and with the vast majority of these studies relying on surrogate markers of mitochondrial content. More recently, a study that has used electron microscopy to evaluate mitochondrial cristae density after prolonged endurance training, suggests that mitochondrial cristae density may provide a stronger predictor of an individual's maximal oxygen uptake compared to mitochondrial volume density (Nielsen et al., 2016). Still, whereas insight on mitochondrial volume density and cristae density can provide relevant information on permanent quantitative changes, such measures are not necessarily telling on adaptations in mitochondrial function.

## Effect of prolonged resistance training on muscle mitochondrial respiratory function

Utilization of high resolution respirometry in permeabillized myofibers and/or isolated mitochondria from muscle biopsies may allow for a more physiologically relevant approach to study mitochondrial function (Haller et al., 1994; Kuznetsov et al., 2008). In this regard, a recent study utilizing high resolution respirometry in permeabilized myofibers observed increased maximal coupled respiration supported by electron transfer from complex 1 and 2 after 12 weeks of high-load resistance training in young, untrained subjects, with only a modest (and non-significant) increase in CS activity (Porter et al., 2015). This implies that the maximal ATP-producing capacity can be improved independently of changes in mitochondrial content. Similarly, by a comparative approach also utilizing high resolution respirometry in permeabilized myofibers, Pesta et al. (2011) found identical magnitudes of improvements in intrinsic mitochondrial function following 10 weeks of high-load resistance training and endurance training undertaken under normoxic as well as hypoxic conditions, although it needs to be emphasized that only three participants completed the normoxic high-load resistance training-intervention. Furthermore, the authors employed measures of mitochondrial DNA as a biomarker, which has elsewhere been contended to constitute a poor predictor of mitochondrial content (Larsen et al., 2012). Further supporting these findings, a cross-sectional study reported higher maximal coupled respiration and tighter coupling of oxidative phosphorylation (suggestive of increased efficiency of the phosphorylation system) in permeabilized myofibers of highly resistance trained subjects compared to untrained controls, despite no differences in CS activity (Salvadego et al., 2013). Such qualitative adaptations may be ascribed simply to increased abundance of electron transport chain complexes following prolonged high-load resistance training (Porter et al., 2015), but may also rely on other contributing mechanisms. Accordingly, recent pioneering studies have provided evidence for increased assemblies of respiratory supercomplexes as well as increased mitochondrial cristae density in response to long term endurance training, thus facilitating qualitative modulation of oxidative capacity (Nielsen et al., 2016; Greggio et al., 2017). Whether similar adaptational changes also adhere to resistance training, remain to be investigated.

Peculiarly, contrary to studies on permeabilized fibers, employment of high resolution respirometry on isolated mitochondria indicates little effect of high-load resistance training on mitochondrial function. In accordance, two recent randomized controlled studies collectively favor the contention that high-load resistance training is not able to augment mitochondrial respiratory function (Irving et al., 2015; Robinson et al., 2017). It can be speculated that the discordance in results between analyses on permeabilized fibers vs. isolated mitochondria stem from organelle interactions inherent of the normal milieu of the muscle cell. Hence, in the living cell, mitochondria exist in fused networks with preserved interactions with other organelles such as the endoplasmic reticulum and the cytoskeleton (Kirkwood et al., 1986; Rizzuto et al., 1998). From this perspective, analysis on permeabilized myofibers can be considered as more closely resembling genuine physiological conditions. Oppositely, mitochondrial isolation procedures can be contended to disrupt the mitochondrial structure and consequently interfere with respiratory function (Picard et al., 2011), but this needs to be further investigated. For comprehensive considerations on the utilization of permeabilized fibers vs. isolated mitochondria, we refer to a previous report (Kuznetsov et al., 2008).

## Summary: effects of resistance exercise on muscle mitochondrial adaptations

The results of relevant resistance exercise studies on mitochondrial biogenesis, content, and function are summarized in Table 1.

**Table 1:** List of included studies on the effect of resistance exercise on mitochondrial adaptations in healthy subjects.

**Reference**	**Duration and frequency**	**Mitochondrial endpoints**			**Comments**
		**Biogenesis**	**Content**	**Function**	
**HIGH-LOAD RESISTANCE EXERCISE—ACUTE STUDIES**					
Wilkinson et al., 2008^*^	Single bout	Unaccustomed-RE ↑	N/A	N/A	Dietary status: fed (protein, carbohydrate, fat)
		Accustomed-RE: ↔			
Donges et al., 2012	Single bout	↑	N/A	N/A	Dietary status: fed (protein)
**HIGH-LOAD RESISTANCE TRAINING—CHRONIC STUDIES**					
MacDougall et al., 1979	24 weeks, 3 days week^−1^	N/A	↓	N/A	
Luthi et al., 1986	6 weeks, 3 days week^−1^	N/A	↓	N/A	
Tesch et al., 1990	12 weeks, 3 days week^−1^	N/A	↔	N/A	Non-exercise control group included
Wang et al., 1993	18 weeks, 2 days week^−1^	N/A	↔	N/A	
Ploutz et al., 1994	9 weeks, 2 days week^−1^	N/A	↔	N/A	
Chilibeck et al., 1999	12 weeks, 3 days week^−1^	N/A	↓	N/A	Non-exercise control group included
Green et al., 1999	12 weeks, 3 days week^−1^	N/A	↔	N/A	
Bell et al., 2000	12 weeks, 3 days week^−1^	N/A	↔	N/A	Non-exercise control group included
Tang et al., 2006	12 weeks, 5 days week^−1^	N/A	↑	N/A	
Wilkinson et al., 2008^*^	10 weeks, 2-3 days week^−1^	N/A	↔	N/A	
Pesta et al., 2011	10 weeks, 3 days week^−1^	N/A	↔	↑	HRR: permeabilized myofibers
Salvadego et al., 2013	Cross-sectional design	N/A	RE-trained vs. untrained: ↔	RE-trained vs. untrained: ↑	HRR: permeabilized myofibers
Kon et al., 2014	8 weeks, 2 days week^−1^	N/A	↓	N/A	
Irving et al., 2015	8 weeks, 4 days week^−1^	N/A	Young: ↔	Young: ↔	Non-exercise control group included
			Aging: ↔	Aging: ↔	HRR: isolated mitochondria
Porter et al., 2015	12 weeks, 2 days week^−1^	N/A	↔	↑	HRR: permeabilized myofibers
Robinson et al., 2017	12 weeks, 4 days week^−1^	Young: ↔	Young: ↑	Young: ↔	Non-exercise control group included
		Aging: ↑	Aging: ↑	Aging: ↔	HRR: isolated mitochondria
					Individual mitochondrial protein content evaluated by proteomics
**LOW-LOAD RESISTANCE EXERCISE—ACUTE STUDIES**					
Burd et al., 2012	Single bout	↑	N/A	N/A	Dietary status: fed (protein)

RE, resistance exercise; HRR, high resolution respirometry;

* *same paper*.

Judged from the ability to increase MitoPS, single-bout low-load as well as high-load resistance exercise can stimulate human muscle mitochondrial biogenesis. However, the influence on resistance exercise-induced MitoPS of training status, resistance exercise principles, and dietary conditions inherent of experimental protocols, deserves further attention. Implementation of non-exercise control experiments and utilization of tracers, allowing for assessment of proteins with slower turnover rate, may substantially aid such investigation.

With regards to accumulated effects of high-load resistance training on permanent mitochondrial adaptations, results remain more equivocal. In accordance, interpretation of studies on mitochondrial content is hampered by evaluation from surrogate markers of mitochondrial content and/or limited sample size. Utilization of electron microscopy combined with sufficient statistical power, would likely allow for more firm conclusions. As judged from the ability to increase mitochondrial respiration, high-load resistance exercise can stimulate mitochondrial function. Yet, since this has so far only been reported from studies on permeabilized fibers, but not confirmed in studies on isolated mitochondria, this specific methodological aspect also requires further attention.

To recapitulate, an accumulating burden of proof favors the contention that high-load resistance training can promote mitochondrial adaptations. Yet, future studies considering some of the factors mentioned above, are warranted before firm conclusion can be made. Furthermore, virtually all previous studies have employed high-load resistance exercise, while still leaving it interesting if low-load resistance exercise can stimulate mitochondrial adaptations.

## Considerations on the utilization of low-load resistance exercise to stimulate muscle mitochondrial adaptations

From a clinical perspective, it is highly interesting that high-load resistance training possess a more pronounced ability to augment mitochondrial adaptations in older adults (Jubrias et al., 2001; Melov et al., 2007; Robinson et al., 2017) and patients with certain chronic disorders (Balakrishnan et al., 2010; Sparks et al., 2013) characterized by mitochondrial abnormalities. This higher consistency than observed in healthy young individuals is likely simply explained by a greater potential for improvement (i.e., lower basal mitochondrial biogenesis inherent of such populations; Rooyackers et al., 1996a,b), which may translate into more robust adaptational changes with prolonged resistance training.

However, whereas current evidence supports that high-load resistance training can stimulate mitochondrial adaptations in clinical populations, high loading may not always be feasible in specific subpopulations. It is therefore interesting that one study by Burd et al. (2012) supports that low-load resistance exercise can stimulate MitoPS. In accordance, the authors observed a robust increase in MitoPS rate after low-load resistance exercise performed with a slow and tonic contraction phase and conducted to volitional fatigue. It is also interesting to note from the study by Burd et al. (2012), that the increased MitoPS rate was observed in subjects with more than 2 years of prior resistance training experience, as this indicates that acute increases in MitoPS rate are not merely to be ascribed a general stress due to lack of exercise habituation.

Fatiguing low-load and/or high-volume resistance exercise has been shown to produce greater ATP turnover compared to traditional high-load resistance exercise (Gorostiaga et al., 2012; Ahtiainen et al., 2015). Furthermore, fatiguing low-load resistance exercise conducted with slow and tonic contraction phase or application of external restriction of blood flow to the exercising muscle, has been shown to impose a more pronounced decrease in tissue oxygenation compared to traditionally performed high- and low-load resistance exercise (Tanimoto and Ishii, 2006; Karabulut et al., 2014; Lauver et al., 2017). It therefore seems plausible that fatiguing low-load resistance exercise entail greater metabolic stress, to potentially accentuate mitochondrial biogenesis.

While traditional low-load resistance exercise performed to volitional fatigue may offer a less mechanically strenuous alternative, it must still be regarded strenuous in terms of time and effort. In this regard, low-load blood flow restricted resistance exercise may offer an alternative in specific cohorts. In accordance, the added ischemia inherent of low-load blood flow restricted resistance exercise has been demonstrated to substantially reduce the time required to reach a point of volitional fatigue and thereby reduce the required work volume, while still stimulating muscle accretion equally much as free-flow low-load resistance exercise performed to fatigue (Farup et al., 2015). On the other hand, blood flow restricted resistance exercise may entail discomfort, especially in the unaccustomed state, so further investigation is required on the feasibility of this approach for clinical populations. Future comparative studies on stimulation of muscle mitochondrial adaptations with fatiguing low-load resistance training (traditional and/or blood flow restricted) vs. high-load resistance training, would be interesting to perform on healthy as well as patient populations.

## Author contributions

All authors listed have made a substantial, direct and intellectual contribution to the work, and approved it for publication.

### Conflict of interest statement

The authors declare that the research was conducted in the absence of any commercial or financial relationships that could be construed as a potential conflict of interest.
